# Analysis of Patient-Physician Concordance in the Understanding of Chemotherapy Treatment Plans Among Patients With Cancer

**DOI:** 10.1001/jamanetworkopen.2020.0341

**Published:** 2020-03-03

**Authors:** Hani Almalki, Ahmed Absi, Abdulrahman Alghamdi, Mohammed Alsalmi, Muhammad Khan

**Affiliations:** 1 College of Medicine, King Saud bin Abdulaziz University for Health Sciences, Jeddah, Saudi Arabia; 2 King Abdullah International Medical Research Center, Jeddah, Saudi Arabia; 3 Department of Medical Oncology, Princess Noorah Oncology Center, King Abdulaziz Medical City, Ministry of the National Guard–Health Affairs, Jeddah, Saudi Arabia

## Abstract

**Importance:**

The treatment plans of patients with cancer involve multiple modalities that oncologists need to explain to patients. One such modality is chemotherapy, in which information about the goals, duration, and expected complications of therapy are considered fundamental to achieving optimal patient understanding. Therefore, effective communication between patients and their treating physicians is important to ensure patient adherence to treatment and achieve better outcomes.

**Objective:**

To investigate the concordance in the understanding of chemotherapy treatment plans between patients and their treating oncologists and to identify the potential factors associated with concordance.

**Design, Setting, and Participants:**

A cross-sectional study was conducted among adult patients (aged >18 years) with cancer who consented to receive chemotherapy between October 4, 2017, and November 8, 2018. The study also included the treating oncologists of patients receiving chemotherapy. An interview-based structured questionnaire was administered in both inpatient and outpatient settings at the Princess Noorah Oncology Center in Jeddah, Saudi Arabia. The demographic data of patients and physicians were obtained from the ARIA Oncology Information System, a chemotherapy-prescribing software database used at the center. Patients who had a personal history of cancer or were unwilling to be involved in the decision-making process were excluded. Data were analyzed from November 15 to December 20, 2018.

**Exposures:**

Patients’ comprehension and concordance with their treating physicians regarding the aspects of the intended treatment plan.

**Main Outcomes and Measures:**

The main outcomes measured were the patient-physician concordance level in the understanding of treatment plans and the identification of patient-related and physician-related factors associated with the level of concordance.

**Results:**

A total of 151 adult patients (77 men [51.0%] and 74 women [49.0%]) were interviewed. Of those, 144 patients (75.5%) were younger than 60 years, and 52 patients (34.4%) had a college or advanced degree. A total of 20 treating oncologists were interviewed, of whom 14 (70.0%) were men and 6 (30.0%) were women. Arabic was the primary language of 19 oncologists (95.0%), and 19 oncologists (95.0%) had medical practice experience outside of Saudi Arabia. Only 20 patients (13.7%) had full concordance with their physicians regarding the aspects of their treatment plans. The remaining 131 patients (86.2%) had discordance with 1 or more aspect of their treatment plans. The most common aspect of discordance was the planned duration of the chemotherapy regimen, with 104 patients (68.4%) having full discordance. Full patient-physician concordance was more likely among patients with college or advanced degrees (χ^2^_1_ = 17.73; *P* < .001) and patients with a family history of cancer (χ^2^_1_ = 15.88; *P* < .001). In addition, older physicians (>40 years) compared with younger physicians (aged 30-40 years) were more likely to achieve higher rates of full patient-physician concordance, whereas older patients (>60 years) were associated with only partial concordance compared with patients younger than 60 years (χ^2^_1_ = 5.84; *P* = .008), with an AR of 2.7.

**Conclusions and Relevance:**

Most patients showed suboptimal understanding of aspects of their chemotherapy treatment plans. More effort and time should be dedicated to enhancing the understanding of chemotherapy plans among patients with lower educational levels. In addition, self-report surveys that evaluate patients’ understanding of their chemotherapy treatment plans should be developed and added to the informed consent process to objectively assess how much a patient understands and to develop a stepwise patient education program that targets patients with the lowest levels of understanding.

## Key Points

**Question:**

Do patients with cancer understand the treatment plans to which they consent?

**Findings:**

In this cross-sectional study of 151 adult patients with cancer and 20 treating physicians only 13.7% of patients had full concordance with their physician regarding aspects of chemotherapy treatment plans. College or advanced degrees and a family history of cancer were associated with better understanding of treatment plans.

**Meaning:**

These findings suggest that more effort and time should be invested in enhancing the understanding of chemotherapy treatment plans among patients with lower educational levels and/or no family history of cancer.

## Teaser

This cross-sectional study investigates patient-physician concordance in the understanding of chemotherapy treatment plans among adult patients with cancer.

## Introduction

The journey of a patient’s treatment starts with effective patient-physician communication. Effective communication is associated with high-quality health care.^1^ Moreover, understanding a patient’s concerns and preferences with regard to treatment options can aid physicians in identifying misconceptions and lead to better medical decisions.^2^ Nevertheless, effective communication between physicians and patients regarding treatment plans is not easily achieved.^3^ This challenge is more pronounced in patients with cancer because of the complexity of care needed.^4^ The treatment plans of patients with cancer involve multiple modalities, for which oncologists need to explain the therapy goals, duration, and expected complications of treatment. Therefore, effective communication between patients with cancer and their treating physicians is important to ensure patient adherence to treatment and achieve better outcomes.

It is also possible to change treatment plans for such patients according to certain factors, such as tumor response to treatment and toxic effects that may arise from chemotherapy.^5,6^ Discordance in the understanding of the treatment plan between patients and their physicians is not uncommon in the Western world. In 1 study that evaluated hospitalized patients’ understanding of their treatment plans, patients perceived themselves to be fully aware, yet they were in disagreement with their physicians on some aspects of the treatment plans. The study assessed the knowledge of patients at hospital discharge, and no more than one-half of them had accurate information about each aspect of their conditions, which included their diagnoses, medications, and adverse effects of treatment.^7^

Patient education in clinical practice has been associated with substantial positive outcomes. Interventional studies that included targeted health education following a comprehensive assessment of patients’ understanding have indicated positive results.^8,9^ It has been reported that therapeutic education is associated with a decrease in the number of hospitalizations for bronchial asthma and diabetic coma as well as a reduction in the number of lower limb amputations.^10^ Other studies have indicated that physicians and trainees frequently overestimate patients' understanding of their treatment plans at hospital discharge and do not recognize patients’ low health literacy.^11,12^ Although only 57.0% of hospitalized patients reported that they understood the potential adverse effects of their medications on discharge, physicians believed that 89% of their patients understood these effects.

To our knowledge, no study has been carried out in Saudi Arabia regarding this topic. The provision of health care in Saudi Arabia has some unique features that may add to this challenge. These features include the cultural understanding of cancer and the stigma surrounding certain types of cancer, the lack of common medical terminology that patients can understand (which may also result from a patient’s lack of health education), and the fact that a substantial number of health care professionals do not speak Arabic.^13^ Furthermore, the strong ties between Saudi family members can add another challenge regarding the expected level of care. The families of patients with terminal cancer usually expect curative treatments even after any realistic hope of a cure is gone.^14^

The goal of this study was to investigate the concordance between patients and their treating oncologists in the understanding of chemotherapy treatment plans and to investigate the potential patient-related and physician-related factors in this concordance.

## Methods

### Study Design and Setting

An interview-based cross-sectional study was conducted at the Princess Noorah Oncology Center in King Abdulaziz Medical City (Jeddah, Saudi Arabia), which has a total capacity of 751 beds and includes medical and surgical departments in addition to the oncology center. This tertiary medical center is one of the major oncology centers in Saudi Arabia and provides care to the Saudi population and other eligible patients in the western region of Saudi Arabia. The oncology center has 108 beds and is mainly composed of 6 departments that include adult medical oncology, gynecological oncology, adult and pediatric hematology and bone marrow transplant, pediatric oncology, radiation oncology, and palliative care. This study followed the Strengthening the Reporting of Observational Studies in Epidemiology (STROBE) reporting guideline for cross-sectional studies, and it was approved by the institutional review board of the King Abdullah International Medical Research Center.

Participants were asked to voluntarily participate in the study. An approved informed consent form designed by the research center was given to all participants before the interview. Participants’ identities were confidential, and each participant was assigned a generic serial number that was not linked to the medical record number. Participants had the right to withdraw from the study at any time. The collected data were kept confidential and protected from any access by a third party. The data were stored and the passwords protected in the workplace computer, to which only the authors had access.

The study used a consecutive sampling method for selecting the participants between October 4, 2017, and November 8, 2018. The data collection method was an interview-based structured questionnaire, for which patients and physicians were interviewed separately. Patients’ responses were compared with physicians’ responses to assess the level of concordance. Data were analyzed from November 15 to December 20, 2018.

### Participants and Sample Size

The study included Saudi adult patients older than 18 years who were scheduled to receive a therapeutic pharmaceutical cancer treatment that required them to sign an informed consent document. Patients who had a personal history of cancer or were unwilling to be involved in the decision-making process were excluded (Figure).

**Figure.**
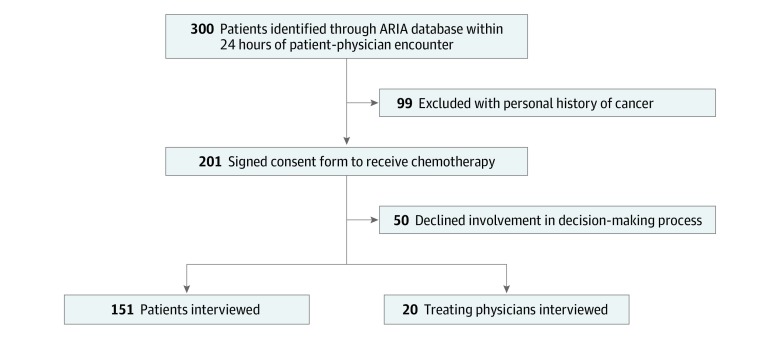
Study Flowchart ARIA indicates ARIA Oncology Information System, which is the chemotherapy-prescribing software database used at the Princess Noorah Oncology Center.

The annual number of patients who receive first-line therapeutic pharmaceutical interventions at the Princess Noorah Oncology Center is approximately 877.^15^ The study used a 95% CI with a 5% margin of error and an assumed response distribution of 50%. The minimum required sample was calculated to be 268 patients.

### Questionnaire

The study used a unified questionnaire as a data collection tool during patient and physician interviews (eMethods in the Supplement). The unified questionnaire ensured that answers were consistently reported during interviews performed by multiple data collectors (H.A., A. Absi, A. Alghamdi, and M.A.). Patients’ and physicians’ interviews were conducted separately.

The questionnaire was composed of 5 parts. The first part addressed the demographic characteristics of each patient and treating oncologist. Patients were asked about their age, sex, educational level, type of cancer, and family history of cancer. Physicians were asked about their age, sex, primary language, nationality, specialty, job title, whether they practiced outside of Saudi Arabia, and whether they practiced in an inpatient or outpatient setting. The second part of the questionnaire comprised the goals of chemotherapy, including curative, palliative, adjuvant, neoadjuvant, and maintenance. The third part evaluated the respondent’s knowledge of the frequency and duration of the chemotherapy cycle. The fourth part measured the respondent’s knowledge of the procedure that would be performed to assess the outcome of treatment via pathological, laboratory, or imaging studies. The fifth part assessed the respondent’s knowledge of the 3 most important toxic effects that were communicated by the physician to the patient.

The questions were multiple choice to ensure consistency in patients’ responses. However, questions about the type of cancer and the frequency of the chemotherapy cycle were open ended and based on the patient’s wording. If the patient’s answer matched the physician’s answer, it was labeled as concordant during the analysis; if it did not match, it was labeled as discordant. Each question had 1 correct answer with the exception of chemotherapy toxic effects, for which the patient had to recall the 3 most important toxic effects explained to them by the treating oncologist.

The demographic data of patients and physicians were obtained from the ARIA Oncology Information System (Varian Medical Systems), a chemotherapy-prescribing software database used at the Princess Noorah Oncology Center. Complementary data were obtained through the interview questionnaire to ensure consistency in responses between patients and physicians.

Patient and physician interviews were conducted separately within 24 hours after obtaining the patient’s consent to receive chemotherapy. During the analysis, patients’ answers were compared with physicians’ answers to measure the level of patient-physician concordance. The content and structure of the questionnaire were validated by an independent biostatistician and 2 oncologists (A. Absi and an independent oncologist). To eliminate the language barrier among patients, the questionnaire was translated into Arabic. A back-translation into English was also performed by a language expert. To ensure the reliability of the questionnaire, a pilot study was conducted on 25 patients. Any confusion or misunderstanding regarding the questions was identified, and the questions were modified appropriately. The final revised version was then used in the study.

### Variables and Bias

The study explored the association between patients’ understanding of their treatment plans and their treating physicians’ sex, age group, primary language, nationality, and practice location (within or outside of Saudi Arabia). The demographic data of patients and physicians were obtained from the ARIA database, and complementary data were obtained through an interview questionnaire to ensure consistency in the responses of patients and physicians.

All variables were qualitative and were coded for storage and analysis using IBM SPSS Statistics, version 23 (IBM SPSS). Qualitative variables were reported as frequencies and percentages. Patient-physician concordance level in therapy goals, duration, outcome, and toxic effects were evaluated based on the patients’ sex, age group, educational level, family history, and type of cancer.

To minimize recall bias, the study included patients who were scheduled to receive a therapeutic pharmaceutical cancer treatment that required them to sign an informed consent document after speaking with their treating physicians. Patient and physician interviews were then conducted separately within 24 hours after the patient signed the chemotherapy consent form. Another potential bias was the fact that data collectors reported the patients’ answers. Therefore, the patient interview was conducted first to blind the data collector to the expected answer that was discussed by the physician.

### Statistical Methods

Analyses of descriptive statistics were performed and reported as proportions and percentages for categorical variables and means (SDs) for continuous variables. Patients’ understanding of treatment plans was evaluated by comparing their answers with those of their treating physicians, and understanding was indicated by the level of concordance between the 2 sets of answers. The overall level of concordance was assessed by the number of correctly answered questions. If all 4 treatment plan–related questions were answered correctly, the patient-physician concordance level was labeled full. If 1 or more questions were answered incorrectly, the concordance was considered partial. Incorrect answers on all questions indicated full discordance.

The association of sociodemographic factors (among patients and physicians) and family history of cancer (among patients) with overall patient-physician concordance in the understanding of the treatment plan was assessed using a χ^2^ test with an analysis of the adjusted residual (AR) and a Fisher exact test. Tests were 2-sided and unpaired, and the statistical significance threshold was *P* < .05.

## Results

A total of 151 adult patients (77 men [51.0%] and 74 women [49.0%]) were interviewed. Of those, 144 patients (75.5%) were younger than 60 years, and 52 patients (34.4%) had a college or advanced degree (Table 1). Twenty treating oncologists were interviewed, of whom 14 (70.0%) were men and 6 (30.0%) were women. Arabic was the primary language spoken by 19 physicians (95.0%), and 19 physicians (95.0%) had practiced medicine at some point outside of Saudi Arabia (Table 2). A total of 87 patients (57.6%) were receiving care from medical oncology teams, and 59 patients (39.1%) were receiving care from hematology teams. Seventy-seven patients (51.0%) were interviewed in an outpatient setting, while the remaining 74 patients (49.0%) were interviewed during their hospitalization. All 20 of the treating physicians participated and were interviewed more than once because they provided care for multiple patients in the study.

**Table 1.** Demographic Characteristics of Patients

Characteristic	No. (%)		
	Total	Male	Female
Total	151 (100)	74 (49.0)	77 (51.0)
Age, y			
18-30	23 (15.2)	10 (13.5)	13 (16.9)
31-60	91 (60.3)	50 (67.5)	41 (53.2)
>60	37 (24.5)	14 (18.9)	23 (29.9)
Educational level			
Less than high school	52 (34.4)	24 (32.4)	28 (36.4)
High school	47 (31.1)	25 (33.8)	22 (28.6)
College or advanced degree	52 (34.4)	25 (33.8)	27 (35.1)
Family history of cancer			
No	109 (72.2)	52 (70.3)	57 (74.0)
Yes	42 (27.8)	22 (29.7)	20 (26.0)

**Table 2.** Demographic Characteristics of Physicians

Characteristic	No. (%)
Total	20 (100)
Sex	
Male	14 (70.0)
Female	6 (30.0)
Age, y	
30-40	9 (45.0)
>40	11 (55.0)
Nationality	
Saudi	14 (70.0)
Non-Saudi	6 (30.0)
Medical practice outside Saudi Arabia	
Yes	19 (95.0)
No	1 (5.0)
Primary language	
Arabic	19 (95.0)
Other	1 (5.0)
Specialty	
Medical oncology	12 (60.0)
Hematology	7 (35.0)
Gynecological oncology	1 (5.0)
Job title	
Consultant/associate consultant	16 (80.0)
Staff physician	4 (20.0)

No patients had complete discordance with their treating physicians on all aspects of their treatment plans. Therefore, patients were divided into 2 groups based on the level of concordance with their physicians. The first group consisted of those with concordance on 1 or more, but not all, aspects. This group represented most of our sample (131 patients [86.2%]). Those who had full concordance with their physicians on all aspects were included in the second group and accounted for 20 patients (13.7%).

As illustrated in Table 3, the highest patient–physician concordance rate was associated with the type of cancer, for which 123 patients (81.5%) correctly identified their primary cancer diagnosis. In contrast, a notable patient-physician discordance was observed in the duration of the chemotherapy regimen, with 104 patients (68.4%) reporting discordance. Of those, 58 patients (55.8%) reported shorter treatment durations than their physicians, and 46 patients (44.2%) reported longer treatment durations. With regard to treatment goals, 103 patients (68.2%) had concordance with their physicians. However, only 32 patients (21.2%) were in concordance with their physicians regarding the 3 most important toxic effects of their chemotherapy plans, and 34 patients (22.4%) could not identify any of the toxic effects of chemotherapy.

**Table 3.** Patient-Physician Concordance on Aspects of Treatment Plan

Treatment Plan Variable	No. (%)
Type of cancer	
Discordance	28 (18.5)
Concordance	123 (81.5)
Goal of therapy	
Discordance	48 (31.8)
Concordance	103 (68.2)
Duration of chemotherapy regimen	
Discordance	104 (68.4)
Concordance	47 (30.9)
Frequency of chemotherapy cycle	
Discordance	68 (45.0)
Concordance	83 (55.0)
Follow-up method	
Discordance	47 (31.1)
Concordance	104 (68.9)
Toxic effects of chemotherapy	
Discordance	34 (22.4)
Partial concordance	
Identified 1 toxic effect	49 (32.5)
Identified 2 toxic effects	36 (23.8)
Full concordance	32 (21.2)

Adjusted residual values were used to identify statistical significance. A statistically significant difference was found between overall patient-physician concordance across different age groups of physicians (χ^2^_1_ = 5.84; *P* = .02). Older physicians (aged >40 years) were more likely to achieve higher rates of full concordance with their patients compared with younger physicians (Table 4). In contrast, patients older than 60 years were more likely to have partial rather than full concordance with their physicians compared with patients younger than 60 years (37 patients vs 0 patients, respectively; χ^2^_1_ = 5.84; *P* = .008), with an AR of 2.7. In addition, the level of overall patient-physician concordance differed significantly across the 3 levels of patient education (χ^2^_1_ = 17.73; *P* < .001). Patients with college or advanced degrees were more likely to have full concordance with their physicians (AR = 4.1), while patients with less than a high school education were more likely to have only partial concordance (AR = 3.0). A family history of cancer was associated with a greater likelihood of full patient-physician concordance (χ^2^_1_ = 15.88; *P* < .001).

**Table 4.** Bivariate Analysis of Association Between Sociodemographic Variables and Level of Patient-Physician Concordance

Sociodemographic Variable	Concordance Level, No. (%)		*P* Value
	Partial	Full	
**Physicians**			
Age, y			
30-40	76 (92.7)	6 (7.3)	.02^a^
>40	55 (79.7)	14 (20.3)	
Sex			
Male	103 (88.0)	14 (12.0)	.40^b^
Female	28 (82.4)	6 (17.6)	
Practiced medicine abroad			
Yes	125 (87.4)	18 (12.6)	.29^b^
No	6 (75.0)	2 (25.0)	
First language			
Arabic	122 (85.9)	20 (14.1)	.27^b^
Other	9 (100)	0	
Nationality			
Saudi	85 (85.0)	15 (15.0)	.37^a^
Non-Saudi	46 (90.2)	5 (9.8)	
Job title			
Consultant/associate consultant	103 (85.8)	17 (14.2)	.34^b^
Assistant consultant	8 (80.0)	2 (20.0)	
Staff physician	20 (95.2)	1 (4.8)	
Specialty			
Medical oncology	79 (85.7)	13 (14.3)	.83^b^
Hematology	52 (88.1)	7 (11.9)	
Gynecological oncology	1 (100)	0	
**Patients**			
Age, y			
18-30	19 (82.6)	4 (17.4)	.008^a^
31-60	75 (82.4)	16 (17.6)	
>60	37 (100)	0	
Sex			
Male	69 (89.6)	8 (10.4)	.29^a^
Female	62 (83.8)	12 (16.2)	
Educational level			
Less than high school	51 (98.1)	1 (1.9)	<.001^a^
High school	43 (91.5)	4 (8.5)	
College or advanced degree	37 (71.2)	15 (28.2)	
Family history of cancer			
No	102 (93.6)	7 (6.4)	<.001^a^
Yes	29 (69.0)	13 (31.0)	

^a^ Data obtained from χ^2^ test.

^b^ Data obtained from Fisher exact test.

## Discussion

This study assessed the level of patients’ understanding of their treatment plans based on the level of patient-physician agreement in a single oncology center. We observed variable levels of discordance depending on the aspect of the treatment plan. There are multiple explanations for this discordance that pertain to either the physicians’ characteristics and the setting of the encounter or the patients’ characteristics and backgrounds.

In our study, 81.5% of patients were able to correctly identify their diagnosis. However, only 13.7% of patients were in full concordance with their respective physicians in all aspects of their chemotherapy plans. In addition, 55.8% of patients were expecting shorter treatment durations than were their physicians. In another study conducted at the Mayo Clinic, patients were not in agreement with their treating physicians in multiple domains, including diagnosis, planned tests and procedures, medications, and expected date of hospital discharge.^7^ Furthermore, the present study demonstrated a patient-physician concordance of 68.2% in treatment goals, which was comparable with 1 study conducted on patients with cancer in which 69% of patients were in agreement with their physicians with regard to treatment goals.^16^ The remaining discordance might be attributed to multiple factors, such as the fact that physicians may have overestimated their patients’ understanding of the treatment plans or may not have recognized their patients’ levels of health literacy.^11^ Furthermore, time constraints and the load of clinical duties might be factors in the level of physicians’ engagement in discussions with patients.

Multiple studies have assessed patients’ understanding of the common adverse effects of treatment plans in different settings. In a study performed in Columbus, Ohio, 135 patients were contacted 2 to 6 weeks after hospital discharge to assess their level of awareness of and adherence to their treatment plans; the results indicated that only 25% of patients knew the common adverse effects of all of their medications.^17^ In our study of patients’ understanding of expected toxic effects, only 21.2% of patients were able to identify the 3 most important adverse effects of their chemotherapy plans. Notably, 22.4% of patients did not know any of the toxic or adverse effects of chemotherapy. An understanding of such factors is important for shared decision-making, which is a good model of health care provision that depends on patients’ comprehension of health care aspects so they are able to express their values and preferences during the decision-making process. This shared decision-making model is being increasingly implemented today.^18^ An integral part of the model is the proper explanation of informed consent to ensure the patients’ understanding of treatment benefits, the potential for serious adverse effects, and the requirements of further diagnostic evaluation.^19^

The educational level of the patient was significantly associated with a better understanding of the treatment plan, which was manifested as a higher patient-physician concordance. Patients who had college or advanced degrees were more likely to agree with their physicians on treatment plans. Educational level is a recognized factor in a patient’s ability to provide informed consent and understand the treatment plan. An increased level of understanding of cancer was observed in those who had a higher level of education.^20^ Therefore, patients with lower levels of education should be given a more detailed explanation of their treatment plans. In addition, other educational strategies can be implemented, such as social media and multimedia awareness campaigns. A study by Chu et al^21^ suggested that 80% of hospitalized patients expressed willingness to participate in health education activity during hospitalization.

Patients with a family history of cancer had an increased level of agreement with their treating physicians on their treatment plans compared with patients without such a history. This increase in the level of agreement could be associated with a patient’s previous experience with the shock of a cancer diagnosis of a family member. A patient’s ability to absorb the emotional shock of a family member’s diagnosis might result in a better acceptance of the diagnosis in oneself. Therefore, collection and documentation of a patient’s family history can be used as a tool to estimate a patient’s understanding of the treatment plan. Patients with a family history of cancer are more likely to agree with their treating physicians, as suggested in our study sample. The association between a family history of cancer and a patient’s understanding of the cancer treatment plan has not been well investigated. However, a patient’s family history can be used to tailor more effective patient-physician communication, and the literature has indicated that patients with life-threatening diagnoses may benefit from the involvement of family members in their diagnosis and treatment plans. Patients who have made shared decisions about treatment have been reported to have better physical and psychological health outcomes.^22^ In addition, shared decision-making is associated with improvements in overall patient adherence to and satisfaction with cancer treatment plans.^23^

### Limitations

This study had several limitations. First, the interviews were conducted within 24 hours after the patient-physician encounter to minimize recall bias. However, patients who were receiving multiple treatment modalities, including chemotherapy, radiotherapy, and surgical interventions, had difficulty recalling different aspects of their treatment plans. Second, this study was performed in a single center. Therefore, larger-scale studies are necessary for a more precise estimation of patient-physician concordance and assessment of the associated factors. A collaborative study with another oncology center would allow further exploration of the potential cultural differences that may help to explain aspects of the results. Third, patient-physician agreement is subject to the constantly evolving nature of cancer, which can necessitate multiple changes in treatment management plans. Future research should be directed at evaluating the association of the patient’s cancer stage and overall prognosis with the patient’s perception and understanding of treatment goals and plans.

## Conclusions

Most of the patients in this study showed a suboptimal understanding of aspects of their chemotherapy plans. Higher educational levels and family histories of cancer were associated with better understanding. More effort and time should be invested in enhancing the understanding of chemotherapy plans among patients with lower educational levels and/or no family history of treatment with such therapies. In addition, a patient self-report evaluation of the understanding of chemotherapy plans could be added to the informed consent process to assess patients’ level of understanding and develop a stepwise patient education program that targets those with the lowest levels of understanding.

## Notes

Supplement. **eMethods.** Physician and Patient Interview Questionnaire
