# Supplementary material for: Analysis of Patient-Physician Concordance in the Understanding of Chemotherapy Treatment Plans Among Patients With Cancer
Source: JAMA Netw Open. 2020 Mar 3;3(3):e200341. doi: 10.1001/jamanetworkopen.2020.0341 (PMC7054829; doi:10.1001/jamanetworkopen.2020.0341)
Supplement: Supplement. — eMethods. Physician and Patient Interview Questionnaire [file jamanetwopen-3-e200341-s001.pdf]

Supplementary Online Content

Almalki H, Absi A, Alghamdi A, Alsalmi M, Khan M. Analysis of patient-physician concordance in the understanding of chemotherapy treatment plans among patients with cancer. *JAMA Netw Open.* 2020;3(3):e200341. doi:10.1001/jamanetworkopen.2020.0341

eMethods. Physician and Patient Interview Questionnaire

This supplementary material has been provided by the authors to give readers additional information about their work.

© 2020 Almalki A et al. *JAMA Network Open*.

eMethods. Physician and Patient Interview Questionnaire

	Physician	Patient
**Demographic information**	- Age: …..<br>- Gender: ☐Male. ☐Female<br>- Practiced medicine outside Saudi Arabia: ☐Yes ☐No<br>- First Language: ☐Arabic ☐others<br>- Nationality: ☐Saudi ☐Others<br>- Specialty:<br>☐Medical oncology<br>☐Hematology<br>☐Gyn/Oncology<br>- Job title:<br>☐Consultant/ Associate<br>☐Assistant Consultant<br>☐Staff physician<br>- Type of cancer: ………….<br>☐In-patient or ☐our-patient setting	- Study number : …………<br>- Age: ….<br>- Gender: ☐Male. ☐Female<br>Education level:<br>☐Less than high school<br>☐High school<br>☐College<br>☐Advanced degree (Master, PhD)<br>- Type of cancer:......................<br>- Family history of cancer (1st degree only): ☐Yes ☐No
**Goal of therapy**	☐ Curative ☐ Palliative<br>☐ Adjuvant ☐ Neoadjuvant<br>☐ Maintenance	The intention of the chemotherapy is:<br>☐ Curative ☐ Palliative<br>☐ Adjuvant ☐ Neoadjuvant<br>☐ Maintenance
**Duration**	- Frequency of the cycle (in weeks): ……........<br>- Duration of the therapy:<br>☐ X < 4 months ☐ 4-6 months<br>☐ 6-12 months ☐ X > 1 year<br>☐ Unlimited	- Frequency of the cycle (in weeks): …………………<br>☐ Right answer<br>☐ Wrong Answer<br>☐ "I don't know"<br>- Duration of the therapy:<br>☐ < 4 months ☐ 4-6 months<br>☐ 6-12 months ☐ > 1 year<br>☐ Unlimited

© 2020 Almalki A et al. *JAMA Network Open*.

**Outcome**	- Disease status follow up is determined by (primary method)?<br>☐ Pathology<br>☐ Laboratory outcome (Tumor markers)<br>☐ Radiological tumor size reduction	- Disease status follow up is determined by (primary method)?<br>☐ Pathology<br>☐ Laboratory outcome (Tumor markers)<br>☐ Radiological tumor size reduction
*** Toxicity**	-Possible Toxicities **<u>(3 most important):</u>**<br><br>o Nervous system<br>o Cardiac and/or Vascular<br>o Respiratory, thoracic and mediastinal<br>o Endocrine<br>o Gastrointestinal<br>o Musculoskeletal and connective tissue<br>o Renal and urinary<br>o Reproductive system and breast<br>o Blood and lymphatic system<br>o Immune system<br>o Congenital, familial and genetic<br>o Ear and labyrinth<br>o Eye<br>o General disorders and administration site conditions<br>o Hepatobiliary<br>o Infections and infestations<br>o Skin and subcutaneous tissue<br>o Injury, poisoning and procedural complications<br>o Metabolism and nutrition<br>o Neoplasms<br>o Pregnancy, puerperium and perinatal<br>o Psychiatric	What are the most important side effects that was discussed by your treating physician?<br><br>o Nervous system<br>o Cardiac and/or Vascular<br>o Respiratory, thoracic and mediastinal<br>o Endocrine<br>o Gastrointestinal<br>o Musculoskeletal and connective tissue<br>o Renal and urinary<br>o Reproductive system and breast<br>o Blood and lymphatic system<br>o Immune system<br>o Congenital, familial and genetic<br>o Ear and labyrinth<br>o Eye<br>o General disorders and administration site conditions<br>o Hepatobiliary<br>o Infections and infestations<br>o Skin and subcutaneous tissue<br>o Injury, poisoning and procedural complications<br>o Metabolism and nutrition<br>o Neoplasms<br>o Pregnancy, puerperium and perinatal<br>o Psychiatric

*Common Terminology Criteria for Adverse Events (CTCAE) [Internet]. Cancer Therapy Evaluation Program. 2016 [cited 26 December 2016]. Available from: https://evs.nci.nih.gov/ftp1/CTCAE/CTCAE_4.03/CTCAE_4.03_2010-06-14_QuickReference_5x7.pdf

© 2020 Almalki A et al. *JAMA Network Open*.
